# Recombination analysis of *Soybean mosaic virus *sequences reveals evidence of RNA recombination between distinct pathotypes

**DOI:** 10.1186/1743-422X-5-143

**Published:** 2008-11-26

**Authors:** Alla G Gagarinova, Mohan Babu, Martina V Strömvik, Aiming Wang

**Affiliations:** 1 Southern Crop Protection and Food Research Centre, Agriculture and Agri-Food Canada, 1391 Sandford St., London, Ontario, N5V 4T3, Canada; 2 Department of Biology, The University of Western Ontario, Biological & Geological Building, 1151 Richmond St., London, Ontario, N6A 5B7, Canada; 3 Department of Plant Science, McGill University, 21111 Lakeshore Rd., Ste. Anne de Bellevue, Québec, H9X 3V9, Canada; 4 Department of Molecular Genetics, The University of Toronto, Toronto, M5S 1A8, Canada

## Abstract

RNA recombination is one of the two major factors that create RNA genome variability. Assessing its incidence in plant RNA viruses helps understand the formation of new isolates and evaluate the effectiveness of crop protection strategies. To search for recombination in *Soybean mosaic virus *(SMV), the causal agent of a worldwide seed-borne, aphid-transmitted viral soybean disease, we obtained all full-length genome sequences of SMV as well as partial sequences encoding the N-terminal most (P1 protease) and the C-terminal most (capsid protein; CP) viral protein. The sequences were analyzed for possible recombination events using a variety of automatic and manual recombination detection and verification approaches. Automatic scanning identified 3, 10, and 17 recombination sites in the P1, CP, and full-length sequences, respectively. Manual analyses confirmed 10 recombination sites in three full-length SMV sequences. To our knowledge, this is the first report of recombination between distinct SMV pathotypes. These data imply that different SMV pathotypes can simultaneously infect a host cell and exchange genetic materials through recombination. The high incidence of SMV recombination suggests that recombination plays an important role in SMV evolution. Obtaining additional full-length sequences will help elucidate this role.

## Findings

*Soybean mosaic virus *(SMV) is a member of the genus *Potyvirus*, the family *Potyviridae*. It is one of the most devastating viral pathogens of soybean crops causing severe symptoms such as mosaic, mottling, chlorosis and rugosity in leaves, as well as reductions in plant growth with yield losses of up to 100% [1-4]. Like all potyviral genomes, the SMV genome is a single-stranded, positive-sense RNA molecule that is approximately 10 kb in length and contains a single open reading frame [4-6]. It encodes a large polyprotein that is co- and post-translationally cleaved into 11 final protein products [4-6]. SMV is found in all soybean-growing regions of the world. In the United States, at least 98 SMV isolates have been documented [7,8]. Based on their differential interactions with SMV resistant cultivars, these isolates are classified into seven distinct strain groups, G1 through G7 [7,8]. Similarly, five (A to E) and eight (Sa to Sh) SMV strains have been reported in Japan and China, respectively [9-11]. Though the pathotypic relationships between the SMV groups in the United States and the strains in China and Japan are not clear, these data clearly suggest a high genetic diversity of SMV.

The two major factors that contribute to the variability and evolution of RNA viruses are mutation introduced by the viral RNA-dependent RNA polymerase and recombination between different viral RNA molecules [12]. The mutation rate varies between virus species, and the recombination frequency is dependent on the degree of sequence similarity between the sequences involved, the length of viral genome and the presence of recombination hot spots [12-14]. Mutation has been demonstrated to be responsible for the emergence of new SMV isolates that differentiate from their parental isolates by breaking resistance in soybean under both field and laboratory conditions [15-17]. However, the role of RNA recombination in SMV evolution still remains unknown.

We evaluated RNA recombination in SMV. All partial SMV sequences encoding the N-terminal most (P1 protease) and the C-terminal most (capsid protein; CP) as well as full-length SMV sequences were retrieved from GenBank [Additional file 1]. The alignments were performed using ClustalW [18]. Subsequently, the automatic recombination scans of the sequence alignments were performed using Recombination Detection Program v.3.31 (RDP3) with default settings [19]. RDP3 scans all possible triplet combinations of sequences to identify and statistically test the recombination signals [19]. When two (parental) sequences are joined to form a recombinant (daughter) sequence, recombination signals may be detected in the parental, daughter, any descendant, and other closely related isolates. Thus, it is possible that a single recombination event can be counted several times. RDP3 overcomes this complication by automatically combining recombination signals to identify a minimum set of unique recombination events that account for the observed similarity patterns among sequences.

**Figure 1:**
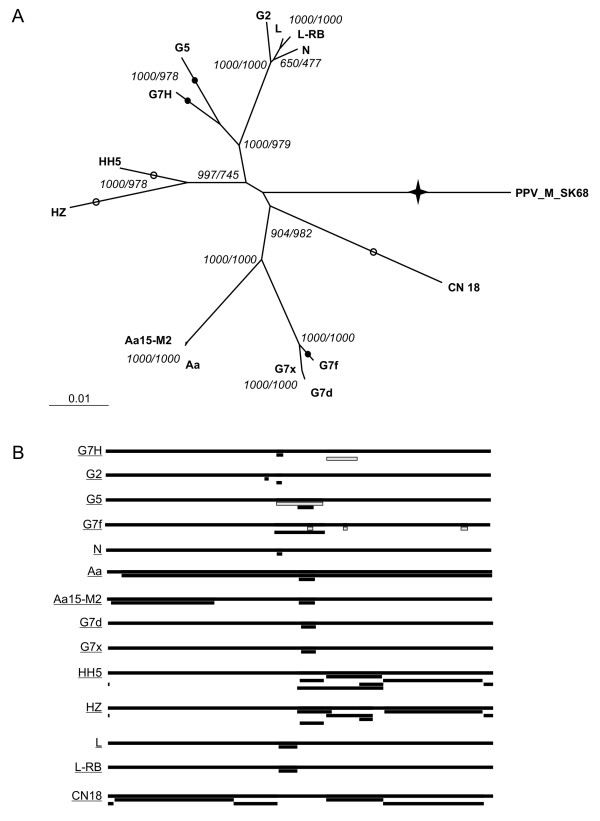
**Recombination in full-length SMV sequences.** A. Phylogenetic relationships of SMV isolates to each other and to PPV as an outgroup. Phylogenetic tree was constructed using full-length nucleotide sequences of isolates L [GenBank: EU871724], L-RB [GenBank: EU871725], G2 [GenBank: S42280.1], N [GenBank: D00507.2], Aa [GenBank: AB100442.1], Aa15-M2 [GenBank: AB100443.1], G5 [GenBank: AY294044.1], G7H [GenBank: AY294045.1], G7d [GenBank: AY216987.1], G7 referred as G7x [GenBank: AY216010.1], and G7 referred as G7f [GenBank: AF241739.1], CN18 [GenBank: AJ619757], HH5 [GenBank: AJ310200], HZ [GenBank: AJ312439], as well as PPV [GenBank: M92280.1], as the outgroup, and the Neighbour Joining function of ClustalX [34]. Topologies of the Bayesian [35] as well as the1000 times bootstrapped least squares [36] and maximum likelihood [37] phylogenetic trees were same (data not shown). Bootstrap values for the Neighbour Joining and the maximum likelihood phylogenetic trees, out of 1000 replicates, are given at the nodes before and after the slanted line, respectively. For presentation purposes, the line marked with a star was shortened from 0.35145 to 0.04145. Automated RDP3 recombination analysis identified recombination events in all SMV isolates [please also see Additional file 1]. Filled circles demarcate likely times, when in evolution of SMV manually verified recombination events took place, while empty circles demarcate significant (*P*-value < 0.05) recombination events where the likely recombinant isolates were determined to be too far diverged from all available SMV sequences for the χ^2 ^analysis of recombination and thus recombination analyses results were considered inconclusive. B. Locations of unique recombination events identified by RDP3, in relation to the full-length sequence alignment [please also see the Additional file 1]. Each full-length genome is represented by a long black bar and the corresponding underlined isolate name, given to the left of the bar. The figure shows a total of 17 unique recombination events, demarcated by the bars below the genomes the recombinant fragments have been integrated into. When an ancestral unique recombination event can be found in more than one daughter sequence, the recombination event is displayed with all corresponding daughter sequences. Locations of the unique recombination events identified by RDP, corresponding to the manually verified recombination sites, are shown with grey bars [please also see Additional file 1].

Our RDP3 analyses identified many unique recombination events in full-length, P1, and CP alignments (Figure 1) [Additional file 2]. However, because of the apparently large number of ancestral and overlapping recombination signals in full-length SMV sequences, final assignments of parental and daughter designations in the identified unique recombination events were affected by the order in which the sequences were analyzed. This ambiguity was likely caused by the limited number of full-length genome sequences for many SMV strains. Manual examination of the RDP3 results did not reveal a better set of the unique recombination events, suggesting the complex similarity patterns among SMV sequences could arise through recombination in diverse ways (data not shown). In accordance with the parsimony principle, we have presented the output that explains the relationships between SMV sequences in all alignments by the smallest number of recombination events (Figure 1B) [Additional file 2].

We further examined more recent, in evolutionary terms, recombination events in full-length SMV sequences. Putative non-recombinant and recombinant sequences were identified using Simplot [20] with 200 bp window and 20 bp step sizes. Location and significance of each putative recombination site was tested using the χ^2 ^test, or the informative sites analysis, implemented in Simplot [21-23]. The recombination site was placed where the highest significant χ^2 ^value was obtained. Two blocks of sequences, on either side of the recombination site, each from a single parent, were compared to assess the likelihood of recombination at the given site. First, non-SMV potyvirus and, subsequently, a distantly related isolate of SMV were used as outgroups in χ^2 ^tests to increase the number of informative sites and to narrow down the location of the recombination. Essentially, locations of the putative recombination sites identified by the Simplot coincided with the locations of the unique recombination events identified by RDP3 (data not shown). Recombination sites in CN18, HZ, and HH5 sequences were supported with a *P*-value < 0.05 by the χ^2 ^test. However, no recombination sites were manually assigned to these isolates since a large number of un-uniformly distributed informative sites supported grouping of these isolates with the outgroup in all tests, indicating that isolates CN18, HZ, and HH5 were too diverged from all other SMV sequences for the χ^2 ^test (data not shown). Nevertheless, a number of significant recombination events were identified in G5, G7H, and G7f sequences.

**Figure 2:**
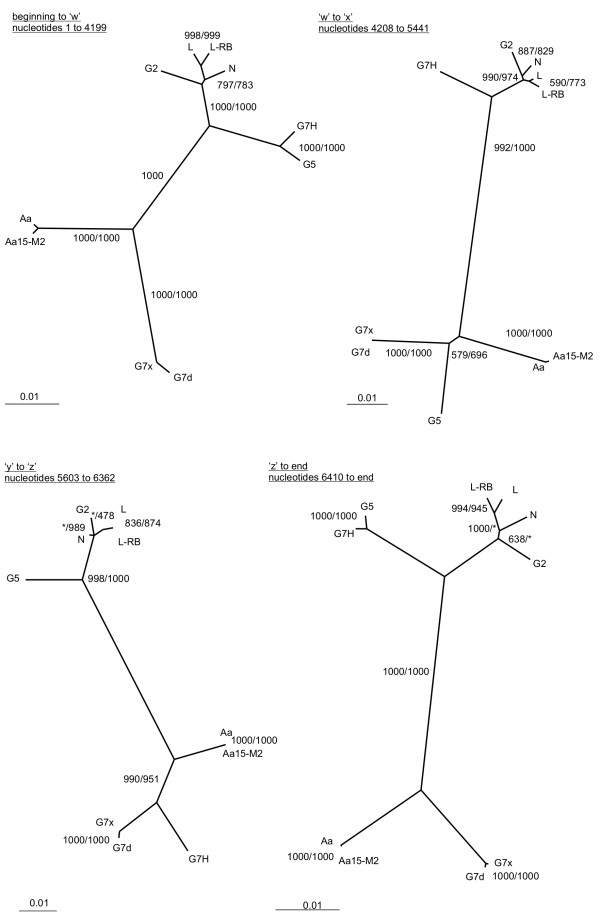
**Phylogenetic trees for the alignment regions demarcated by G5 and G7H recombination sites.** Non-recombinant, as determined by the manual recombination analysis (see manuscript text), as well as G5 and G7H sequences were included in the phylogenetic tree construction. The designations for the fragments are given at the top, to the left of each tree. Bayesian [35] as well as bootstrapped Neighbour Joining [34], least squares [36], and maximum likelihood [37] trees were constructed for each region. Topologies of the trees generated by the four methods for the same region were same, with exception of how C2 and N sequences related to each other and to L and L-RB isolates from recombination site 'y' to the end of the alignment. Shimodaira-Hasegawa (SH) test [38] was used to select the best of the competing but very similar topologies for each sequence region (date not shown). The tree topology that obtained the highest SH score of 1 is presented for each region. Bootstrap values out of 1000 replicates, produced by the Neighbour Joining and the maximum likelihood methods are given at the nodes, before and after the slanted line, respectively. A star given instead of the number indicates that the respective method did not agree with the topology of the optimal tree identified by the SH test at that particular node. Topologies of all trees were tested against each other and the topologies of the trees presented here were found optimal (SH score: 1). SH scores of 0 were obtained when topologies of the trees from between recombination sites were tested against sequence alignments for the regions on the basis of which the given tree was not generated. The same SH score of 0 was obtained when the tree topologies for the regions from the beginning of the sequence alignment to 'w' and from 'z' to end of the sequence alignment were tested against sequence alignments between the recombination sites. Collectively, these results indicated that the different topologies cannot substitute for each other in explaining the variability of SMV sequences between G5 and G7H recombination sites that we identified.

Two SMV isolates, G5 and G7H, though belonging to two different pathotypes, were previously reported to be closely related to each other based on full-length genome sequence comparison [24]. Consistently with this finding, isolate G7H indeed clustered with isolate G5 and not with pathotype G7 isolates in a phylogenetic tree constructed from the full-length SMV genome sequences (Figure 1A). Recombination sites, 'w', 'x' and 'z' in G5 and 'w', 'y', and 'z' in G7H were supported statistically with a *P*-value < 0.05 by the χ^2 ^test. Following were the locations of these sites in respect to the G5 and G7H sequences: 'w' 4199 – 4208, 'x' 5441 – 5546, 'y' 5546 – 5603, and 'z' 6362 – 6410. Similarity patterns and phylogenetic trees constructed for the sequence alignment regions demarcated by the recombination sites confirmed two recombination events in each of the isolates: 'w' and 'x' in G5 and 'y' and 'z' in G7H (Figure 2) [Additional file 3] [Additional file 4]. These recombination sites in G5 and G7H were supported statistically with a *P*-value < 0.001 [Additional file 3] [Additional file 4]. These phylogenetic and recombination analysis results suggest that majority of G5 and G7H genome sequences were derived from a common ancestor more closely related to the G2 group of isolates, while fragments between recombination sites 'w' and 'x' in G5 and 'y' and 'z' in G7H are more closely related to G7x and G7d (Figure 2). The high-confidence phylogenetic grouping of G7H with G7d and G7x is consistent with the location of factors distinguishing G7 pathotype from G2 and G5 pathotypes somewhere between recombination sites 'y' and 'z', in the genome region encoding the C-terminal part of 6K2-VPg and the N-terminal part of NIa-Pro.

Analyses of the G7f genome revealed six recombination events, 'a' through 'f', that occurred in the formation of this isolate. G7f was most similar to G7x and G7d isolates along most of its genome, but was more similar to G2 between recombination sites 'a' and 'b', 'c' and 'd', 'e' and 'f' [Additional file 5]. The following were the nucleotide position numbers of recombination event locations in respect to the G7f sequence: 'a' 5102–5114, 'b' 5252–5285, 'c' 6021–6026, 'd' 6140–6176, 'e' 8846–8858, 'f' 9008–9035. In χ^2 ^tests, all 6 recombination sites were supported with *P*-value < 0.0025. However, either mutation, coupled with strong selection, or recombination could result in an isolate being most similar to two different isolates in the neighbouring regions [25]. Selection of mutations would be expected to act at the amino acid sequence level as, to the best of our knowledge, avirulence determinants have only been reported to act at this level [26-32]. On the other hand, recombination may or may not affect the amino acid sequence of the resulting chimera. In this study, most sites that supported grouping of G7f with G2 were silent [Additional file 6] [Additional file 7], providing support for recombination rather than selection hypotheses to explain these similarities. All manually identified G5, G7H, and G7f recombination sites were recapitulated by manual GENECONV test implemented in RDP3 (data not shown).

The manually generated and verified results presented here provide the strong evidence of recombination in SMV. The most parsimonious output of RDP3 for full-length sequences [Additional file 2] partially, but better than other RDP3 outputs, coincided with the manual sequence comparisons results, emphasizing the need for the manual verification of automatically generated results. Furthermore, the G5 and G7H recombination analysis results suggest recombination analysis as a tool for directing experiments to identify avirulence determinants and develop novel crop protection strategies. However, utility of recombination detection in SMV is still limited by the lack of representative full-length genome sequences for most of SMV strains. Availability of representative sequences with associated pathogenicity profiles will allow elucidating the evolutionary history of SMV and deriving testable hypotheses about SMV-soybean interactions.

In spite of limitations to analyzing SMV recombination, application of conceptually different, complementary approaches allowed us to detect recombination sites previously missed by Chare and Holmes [33]. Our work showed, for the first time, that recombination occurred during SMV evolution among distinct viral isolates and thus provided evidence that at least two distinct viral SMV pathotypes can simultaneously infect a host cell and exchange genetic materials through RNA recombination. The high frequency of recombination detected in SMV suggests that recombination plays an important role in SMV evolution and this should be considered when novel antiviral strategies are developed.

## Competing interests

The authors declare that they have no competing interests.

## Authors' contributions

AGG acquired SMV genomic sequences and performed the analysis. AGG, MB, MVS, and AW interpreted the data. AW conceived the study. AGG and AW wrote the paper. All authors critically reviewed and approved the final manuscript.

## Supplementary Material

Additional File 1 **List of full-length and partial (P1, CP) sequences of SMV analysed for recombination.** A list of all sequences and the corresponding Genbank accession numbers are provided. Click here for file

Additional File 2 **Summary of unique recombination events identified by the Recombination Detection Program v.3.31 (RDP3).** Our RDP3 automated analyses using RDP, GENECONV, Bootscan, MaxChi, Chimera, and SiScan methods [19] identified many highly significant recombination signals in full-length, P1, and CP alignments [please see Additional file 1 for the list of accession numbers for all analyzed sequences]. However, when two (parental) sequences are joined to form a recombinant (daughter) sequence, recombination signals will be detected in all descendants of the parental and daughter isolates as well as related sequences, provided the recombination signals have not been obscured by subsequent recombination events or strong selection. All detected recombination signals were automatically combined by RDP3 into sets of unique recombination events. The final set of the unique recombination events depended on the order in which the sequences were analyzed. This effect of sequence analysis order on the generated set of unique recombination events was particularly strong for the full-length sequences, where a large number of ancestral and overlapping recombination signals were found. This ambiguity was likely increased by the lack of full-length genome sequences representing many of the SMV strains. Manual investigation of the RDP3 results did not suggest that any one set of the unique recombination events was better than another: the complex similarity patterns between SMV sequences could arise through recombination in a number of ways (data not shown). Therefore, in accordance with the parsimony principle, we presented the output that explains the relationships between SMV isolates by the smallest number of recombination events. The largest number of unique recombination events was consistently detected by RDP3 in full-length sequences despite the fact that the smallest number of these sequences was analyzed. This may have to do with the fact that complete evolutionary history is preserved in full-length sequences, but not the partial sequences such as P1 and CP that were also analyzed here. More full-length SMV sequences must be obtained in order to describe the broad picture of how recombination affected evolution of SMV. Obtaining additional sequences will also aid in resolving uncertainties about parental and daughter isolate identities and narrowing down the locations of undetermined break points (recombination sites). Click here for file

Additional File 3 **Supplemental Figure 1.** Similarity plots with G5 as the query isolate. Lists of isolates included in the analyses with their corresponding line colors are shown in the legend box. Locations of sites 'w', 'x', 'y', and 'z' are demarcated with vertical lines and the green underlined letters. Regions used for "find sites" analyses are marked with rectangles; names for the query, first and second parental, as well as outgroup isolates, with respective numbers of informative sites, supporting each grouping, and the χ^2 ^values are given for each recombination site in matching colors. Click here for file

Additional File 4 **Supplemental Figure 2.** Similarity plots with G7H as the query isolate. Lists of isolates included in the analyses with their corresponding line colors are shown in the legend box. Locations of sites 'w', 'x', 'y', and 'z' are demarcated with vertical lines and the green underlined letters. Regions used for "find sites" analyses are marked with rectangles; names for the query, first and second parental, as well as outgroup isolates, with respective numbers of informative sites, supporting each grouping, and the χ^2 ^values are given for each recombination site in matching colors. Click here for file

Additional File 5 **Supplemental Figure 3.** Similarity plot with G7f as the query isolate. List of isolates included in the analysis with their corresponding line colors are shown in the legend box. Location of each statistically significant recombination site is demarcated with a vertical line and a green underlined letter, adjacent to the line. Names for the query, first and second parental isolates, and outgroups with corresponding numbers of informative sites, supporting each grouping are given in red and blue, respectively. The χ^2^values for each recombination site are given in italicized underlined font, adjacent to the vertical line demarcating each respective recombination site. Click here for file

Additional File 6 **Supplemental Table 1.** Effect of informative site nucleic acid differences on amino acid composition in analyses of G7f recombination events with remotely related non-SMV potyvirus sequence (PPV) as outgroup. Informative sites that are also found in analyses with Aa as outgroup [see Additional file 7] are given in italics. Click here for file

Additional File 7 **Supplemental Table 2.** Effect of informative site nucleic acid differences on amino acid composition in analyses of G7f recombination events with Aa as outgroup. Informative sites that are also found in analyses with non-SMV potyvirus sequence (PPV) as outgroup [see Additional file 6] are given in italics. Click here for file
