# Supplementary material for: Recombination analysis of Soybean mosaic virus sequences reveals evidence of RNA recombination between distinct pathotypes
Source: Virol J. 2008 Nov 26;5:143. doi: 10.1186/1743-422X-5-143 (PMC2627826; doi:10.1186/1743-422X-5-143)
Supplement: Additional File 1 — List of full-length and partial (P1, CP) sequences of SMV analysed for recombination. A list of all sequences and the corresponding Genbank accession numbers are provided. [file 1743-422X-5-143-S1.pdf]

GenBank accession numbers for P1 sequences:

AJ639655	AJ639654	AJ639653	AJ639652	AJ639651	AJ639650
AJ639649	AJ639648	AJ639647	AJ639646	AJ558194	AJ290450
AJ628762	AJ628761	AJ628760	AJ628759	AJ628758	AB085899
AF200582	AF200579	AF200576	AF200570	AF200567	AF200564
AF200561	AF200558	AF200555	AF200550	AF200547	AF200544
AF200541	AF200538	AF200535			

GenBank accession numbers for CP sequences:

X96665	X63771	AB206834	AB206833	AB206832	AB206831
AB206830	AB206829	AB206828	AB206827	DQ517432	DQ517431
DQ517430	DQ517429	DQ517428	DQ517427	E01309	AB18149
AB181492	AY799852	AJ609298	AY216489	AY216487	AY216485
AY216483	AY216481	AY216479	AB100448	AB100447	AB100446
AB100445	AB100444	AB085900	AF200584	AF200581	AF200578
AF200572	AF200569	AF200566	AF200563	AF200560	AF200557
AF200554	AF200552	AF200549	AF200546	AF200543	AF200540
AF200537	D88616	D88615			

Full-length sequences analyzed for recombination:

L [GenBank: EU871724]	L-RB [GenBank: EU871725]
G2 [GenBank: S42280.1]	N [GenBank: D00507.2]
Aa [GenBank: AB100442.1]	Aa15-M2 [GenBank: AB100443.1]
G5 [GenBank: AY294044.1]	G7 referred as G7f [GenBank: AF241739.1]
G7d [GenBank: AY216987.1]	G7 referred as G7x [GenBank: AY216010.1]
G7H [GenBank: AY294045.1]	CN18 [GenBank: AJ619757]
HH5 [GenBank: AJ310200]	HZ [GenBank: AJ312439]
