# Supplementary material for: Recombination analysis of Soybean mosaic virus sequences reveals evidence of RNA recombination between distinct pathotypes
Source: Virol J. 2008 Nov 26;5:143. doi: 10.1186/1743-422X-5-143 (PMC2627826; doi:10.1186/1743-422X-5-143)
Supplement: Additional File 2 — Summary of unique recombination events identified by the Recombination Detection Program v.3.31 (RDP3). Our RDP3 automated analyses using RDP, GENECONV, Bootscan, MaxChi, Chimera, and SiScan methods [19] identified many highly significant recombination signals in full-length, P1, and CP alignments [please see Additional file 1 for the list of accession numbers for all analyzed sequences]. However, when two (parental) sequences are joined to form a recombinant (daughter) sequence, recombination signals will be detected in all descendants of the parental and daughter isolates as well as related sequences, provided the recombination signals have not been obscured by subsequent recombination events or strong selection. All detected recombination signals were automatically combined by RDP3 into sets of unique recombination events. The final set of the unique recombination events depended on the order in which the sequences were analyzed. This effect of sequence analysis order on the generated set of unique recombination events was particularly strong for the full-length sequences, where a large number of ancestral and overlapping recombination signals were found. This ambiguity was likely increased by the lack of full-length genome sequences representing many of the SMV strains. Manual investigation of the RDP3 results did not suggest that any one set of the unique recombination events was better than another: the complex similarity patterns between SMV sequences could arise through recombination in a number of ways (data not shown). Therefore, in accordance with the parsimony principle, we presented the output that explains the relationships between SMV isolates by the smallest number of recombination events. The largest number of unique recombination events was consistently detected by RDP3 in full-length sequences despite the fact that the smallest number of these sequences was analyzed. This may have to do with the fact that complete evolutionary history is preserved [file 1743-422X-5-143-S2.pdf]

Summary of unique recombination events identified by the Recombination Detection Program v.3.31 (RDP3)

RDP, GENECONV, Bootscan, Maxchi, Chimera, and SiScan, implemented in RDP3, were used for the automated scanning of SMV sequence alignments.

I. Results summary for P1 sequence alignment recombination analysis.

Isolates are identified by their respective Genebank accession numbers (please see Additional file 1 for the list of all analyzed isolates).

Event number	Breakpoint position in recombinant sequence		Recombinant Sequence(s)	Parental sequence(s)		Detection methods implemented in RDP3 (see RDP3 reference for details)					
	**Begin**	**End**		**Minor**	**Major**	**RDP**	**GENECONV**	**Bootscan**	**Maxchi**	**Chimaera**	**SiSscan**
1 (1)	23*	351	AJ628758	AJ628759<br>AJ628761<br>AJ628760	AJ628762	2.00E-07	4.11E-05	1.05E-04	8.38E-08	6.91E-09	1.24E-16
2 (2)	156	334	AF200538<br>AF200555<br>AF200544	AF200535	AJ639654<br>AJ639653<br>AJ639651<br>AJ639647<br>AJ558194<br>AF200567<br>AF200564<br>AF200561	NS	NS	NS	NS	NS	2.39E-05
3 (3)	547	727	AF200535<br>AF200541	AF200550	AF200567<br>AJ639653<br>AJ639647<br>AF200564	NS	NS	NS	NS	NS	1.44E-03

* = The actual breakpoint position is undetermined (it was most likely overprinted by a subsequent recombination event).

Minor Parent = Parent contributing the smaller fraction of sequence.

Major Parent = Parent contributing the larger fraction of sequence.

Unknown = Only one parent and a recombinant need be in the alignment for a recombination event to be detectable. The sequence listed as unknown was used to infer the existance of a missing parental sequence.

NS = No significant P-value was recorded for this event using this method.

II. Results summary for CP sequence alignment recombination analysis.

Isolates are identified by their respective Genebank accession numbers (please see Additional file 1 for the list of all analyzed isolates).

Event number	Breakpoint position in recombinant sequence		Recombinant Sequence(s)	Parental sequence(s)		Detection methods implemented in RDP3 (see RDP3 reference for details)					
	**Begin**	**End**		**Minor**	**Major**	**RDP**	**GENECONV**	**Bootscan**	**Maxchi**	**Chimaera**	**SiSscan**
1 (5)	2048	2603*	X96665	DQ517429<br>DQ517432<br>DQ517427<br>AY216485<br>AY216481<br>AF200584<br>AF200557<br>AF200552<br>AF200546<br>AF200540<br>AF200537	AY216483<br>X63771<br>DQ517431<br>DQ517430<br>DQ517428<br>AY216489<br>AY216487<br>AY216479<br>AB100448<br>AB100447<br>AB100445<br>AB100444<br>AB085900<br>AF200581<br>AF200578<br>AF200572<br>AF200563<br>AF200560<br>AF200554<br>AF200549<br>AF200543<br>D88615	4.08E-04	6.06E-03	1.76E-04	3.38E-05	5.17E-04	5.35E-09
2 (8)	101	414	AB206827	DQ517429	AB181493	NS	NS	NS	NS	NS	8.90E-04

			AB206829<br>AB206828<br>AB100446<br>D88616	AF200537	AB206834<br>AB206833<br>AB206832<br>AB206831<br>AB206830<br>AJ609298						
3 (9)	84	599	AF200566<br>AB206834<br>AB206833<br>AB206832<br>AB206831<br>AB206830<br>AB181493<br>AY799852<br>AJ609298<br>AF200569	AF200563<br>AY216487<br>AY216483	AY216481<br>AY216485<br>AF200584	NS	NS	NS	3.42E-05	2.56E-03	4.86E-04
4 (10)	541	795*	AB100444	AB181492	AB100447	NS	NS	NS	NS	9.73E-03	NS

* = The actual breakpoint position is undetermined (it was most likely overprinted by a subsequent recombination event).
Minor Parent = Parent contributing the smaller fraction of sequence.
Major Parent = Parent contributing the larger fraction of sequence.
Unknown = Only one parent and a recombinant need be in the alignment for a recombination event to be detectable.
The sequence listed as unknown was used to infer the existance of a missing parental sequence.
NS = No significant P-value was recorded for this event using this method.

III. Results summary for the recombination analysis of of full-length sequence alignment.
Isolates are identified by their names, as used throughout the manuscript (please see Additional file 1 for accession numbers and the list of all analyzed isolates).

Event number	Breakpoint position in recombinant sequence		Recombinant Sequence(s)	Parental sequence(s)		ed in RDP3 (see RDP3 reference for details)					
	Begin	End		Minor	Major	RDP	GENECONV	Bootscan	Maxchi	Chimaera	SiSscan
1 (1)	6959	9335	HZ<br>HH5	Unknown (CN18)	L<br>G7H<br>G2<br>G5<br>N<br>L-RB	9.57E-42	5.34E-43	1.02E-47	9.79E-23	2.94E-21	1.61E-19
**2 (2)**	**4297 ('w', #**	**5484 ('x')**	**G5**<br>HH5<br>HZ	G7d<br>G7f<br>Aa<br>Aa15-M2<br>G7x	G7H<br>G2<br>N<br>HH5<br>HZ<br>L<br>L-RB	4.81E-33	6.90E-10	4.72E-32	5.25E-15	1.51E-16	2.93E-12
**3 (3)**	**5581* ('y')**	**6351 ('z', #)**	**G7H**<br>HH5<br>HZ	CN18<br>G7f<br>Aa<br>Aa15-M2<br>G7d<br>G7x<br>HH5	G5<br>G2<br>N<br>L<br>L-RB	1.82E-25	4.35E-17	1.14E-24	1.58E-12	2.34E-12	3.63E-16
4 (4)	6972	9350	CN18	L<br>G7H<br>G2<br>G5<br>N<br>L-RB	Aa<br>G7f<br>Aa15-M2<br>G7d<br>G7x	7.21E-22	1.99E-09	1.19E-17	1.79E-13	3.49E-15	7.90E-18
5 (5)	3148	4290	CN18	Aa<br>G7f	HH5	8.47E-14	1.09E-06	1.51E-13	5.21E-13	8.63E-10	6.68E-17

				Aa15-M2<br>G7d<br>G7x							
6 (6)	0*	109	CN18	Unknown (G5)<br>Unknown(G7H)<br>Unknown(G2)<br>Unknown(N)<br>Unknown(Aa)<br>Unknown(Aa15-M2)<br>Unknown(HH5)<br>Unknown(HZ)<br>Unknown(L)<br>Unknown(L-RB)	G7d<br>G7f<br>Aa<br>Aa15-M2<br>G7x	4.49E-10	3.86E-13	2.23E-07	1.14E-03	1.02E-02	NS
7 (7)	47*	2681	Aa<br>Aa15-M2	L<br>G7H<br>G2<br>G5<br>N<br>HH5<br>HZ<br>L-RB	G7d<br>G7f<br>G7x	1.46E-07	NS	NS	4.46E-10	7.31E-11	6.16E-21
8 (8)	4862	5468*	HH5<br>G5<br>G7f<br>Aa<br>Aa15-M2<br>G7d<br>G7x<br>HZ	CN18<br>G5<br>Aa15-M2	G7H<br>G2<br>N<br>L<br>L-RB	1.30E-04	7.93E-05	1.53E-09	2.97E-09	7.77E-10	1.77E-10
9 (9)	5554*	6971*	CN18<br>HH5<br>HZ	G7d<br>G7f<br>Aa<br>Aa15-M2<br>G7x	N<br>G7H<br>G2<br>L<br>L-RB	6.32E-11	6.52E-07	8.99E-05	2.41E-08	7.48E-08	3.74E-10
10 (10)	163*	3147*	CN18	G7H	Unknown (L)<br>Unknown(G2)<br>Unknown(N)<br>Unknown(L-RB)	NS	NS	2.78E-02	7.52E-10	1.17E-06	3.03E-09
**11 (11)**	**8852 ('e')**	**9021 ('f')**	**G7f**	N<br>G2<br>L<br>L-RB	G7x<br>Aa<br>Aa15-M2<br>G7d	NS	1.40E-06	1.26E-07	NS	NS	NS
12 (12)	4933*	4951*	G2	Unknown (CN18)<br>Unknown(G7f)<br>Unknown(Aa)<br>Unknown(Aa15-M2)<br>Unknown(G7d)<br>Unknown(G7x)	L<br>G7H<br>N<br>L-RB	NS	3.93E-07	NS	NS	NS	NS
13 (13)	9342*	25*	HH5<br>HZ	Unknown (Aa)<br>Unknown(Aa15-M2)	N<br>G2<br>G5<br>L<br>L-RB	1.99E-02	NS	4.52E-04	5.16E-03	7.62E-03	NS
**14 (14)**	**5108* ('a')**	**5258 ('b')**	**G7f**	G2<br>G7H	G7x<br>Aa	NS	4.75E-04	2.91E-03	3.40E-03	3.34E-03	NS

				N<br>L<br>L-RB	Aa15-M2<br>G7d						
**15 (15)**	**6024 ('c')**	**6147 ('d')**	**G7f**	N<br>G2<br>L<br>L-RB	G7x<br>Aa<br>Aa15-M2<br>G7d	NS	9.27E-03	1.44E-03	NS	NS	NS
16 (16)	4012	4097	G2	G7d<br>G7f<br>Aa<br>Aa15-M2<br>G7x	N<br>L	NS	3.41E-03	NS	NS	NS	NS
17 (17)	4296*	4481	G7H<br>G2<br>N<br>L<br>L-RB	Unknown (CN18)	Aa15-M2<br>Aa	3.77E-02	NS	1.60E-02	NS	NS	2.86E-03

* = The actual breakpoint position is undetermined (it was most likely overprinted by a subsequent recombination event).
Minor Parent = Parent contributing the smaller fraction of sequence.
Major Parent = Parent contributing the larger fraction of sequence.
Unknown = Only one parent and a recombinant need be in the alignment for a recombination event to be detectable.
The sequence listed as unknown was used to infer the existance of a missing parental sequence.
NS = No significant P-value was recorded for this event using this method.

Notes on manual verification of recombiantion events in full length sequences:
Manually verified recombination events are shown in bold font (events 2, 3, 11, 14, 15)
Notation, used in the manuscript, for the recombination sites identified manually is indicated within brackets for each recombination event.
# = small disagreement between the location of recombination site found manually and by RDP3.
Analysis of specific sites supporting recombination events supported the recombinaiton event positions determined in manual analyses.

The RDP listed all potential parental and recombinant sequences (i.e. all sequences in which recombination signature was apparent). The most likely pattern of recombination, including the most likely parental isolates or isolate groups, was detected by manual recombination analysis. This analysis also took into consideration the histories of the isolates in determining the identities of parental sequences. For instance, the same informative sites supported G2 and N as the minor parent of G7f between its recombination sites. However, because sequencing of isolates G2 and G7f was reported at the same time and the isolates at some point may have come into contact, we proposed G2 as the minor parent of G7f. However, isolate N was also a possible parent. Conclusions about parental isolate identities thus often apply to the origin o the sequence fragments on the level of isolate groups. Precise identification of the parental isolate sequences requires additional information than what was available for our analysis and a controlled environment where only certain isolates are allowed to come into contact, which is not what usually occures in the field.
