# Supplementary figures and images for: Recombination analysis of Soybean mosaic virus sequences reveals evidence of RNA recombination between distinct pathotypes
Source: Virol J. 2008 Nov 26;5:143. doi: 10.1186/1743-422X-5-143 (PMC2627826; doi:10.1186/1743-422X-5-143)

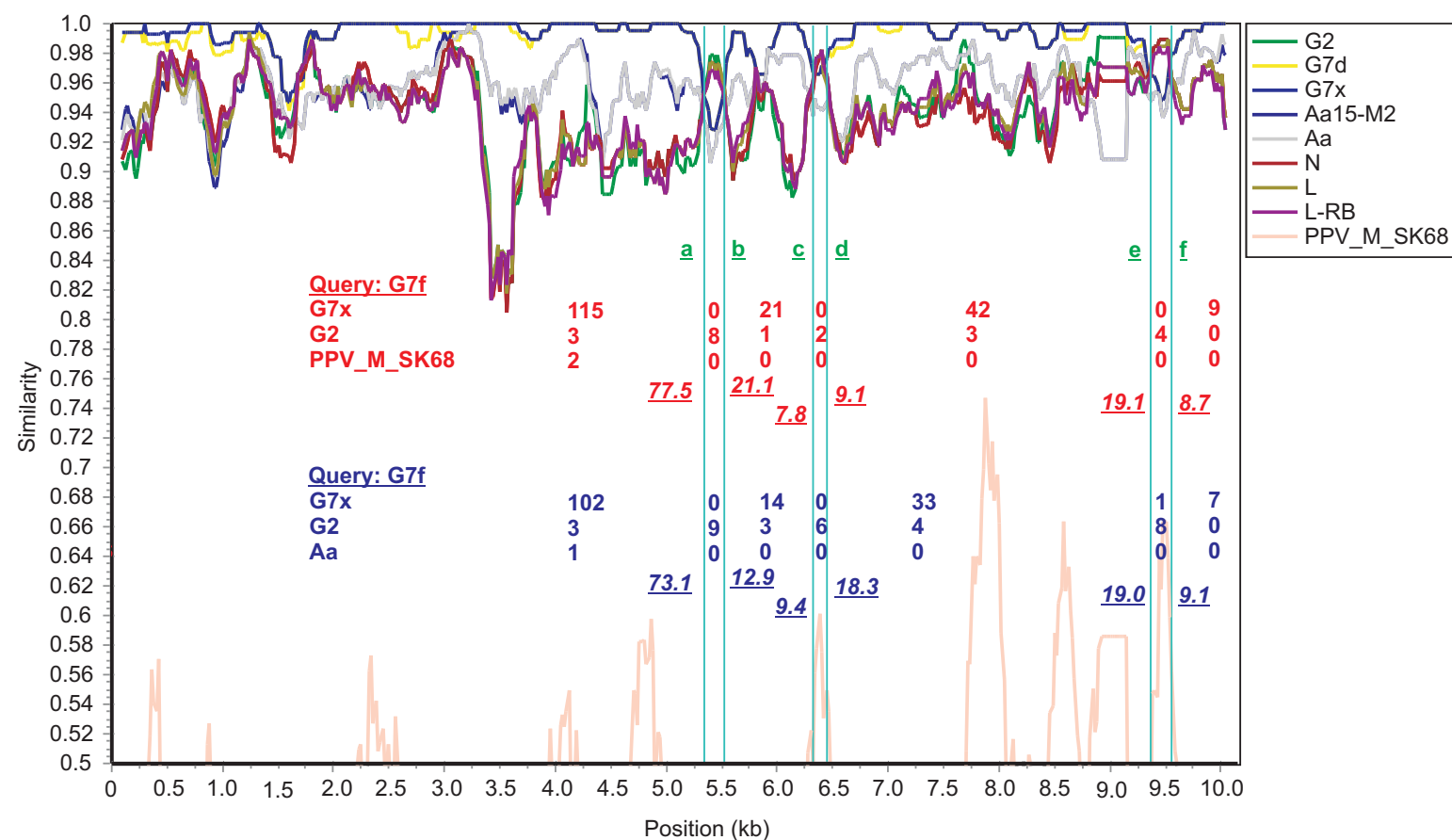

Supplement: Additional File 3 — Supplemental Figure 1. Similarity plots with G5 as the query isolate. Lists of isolates included in the analyses with their corresponding line colors are shown in the legend box. Locations of sites 'w', 'x', 'y', and 'z' are demarcated with vertical lines and the green underlined letters. Regions used for "find sites" analyses are marked with rectangles; names for the query, first and second parental, as well as outgroup isolates, with respective numbers of informative sites, supporting each grouping, and the χ2 values are given for each recombination site in matching colors. [file 1743-422X-5-143-S3.pdf]

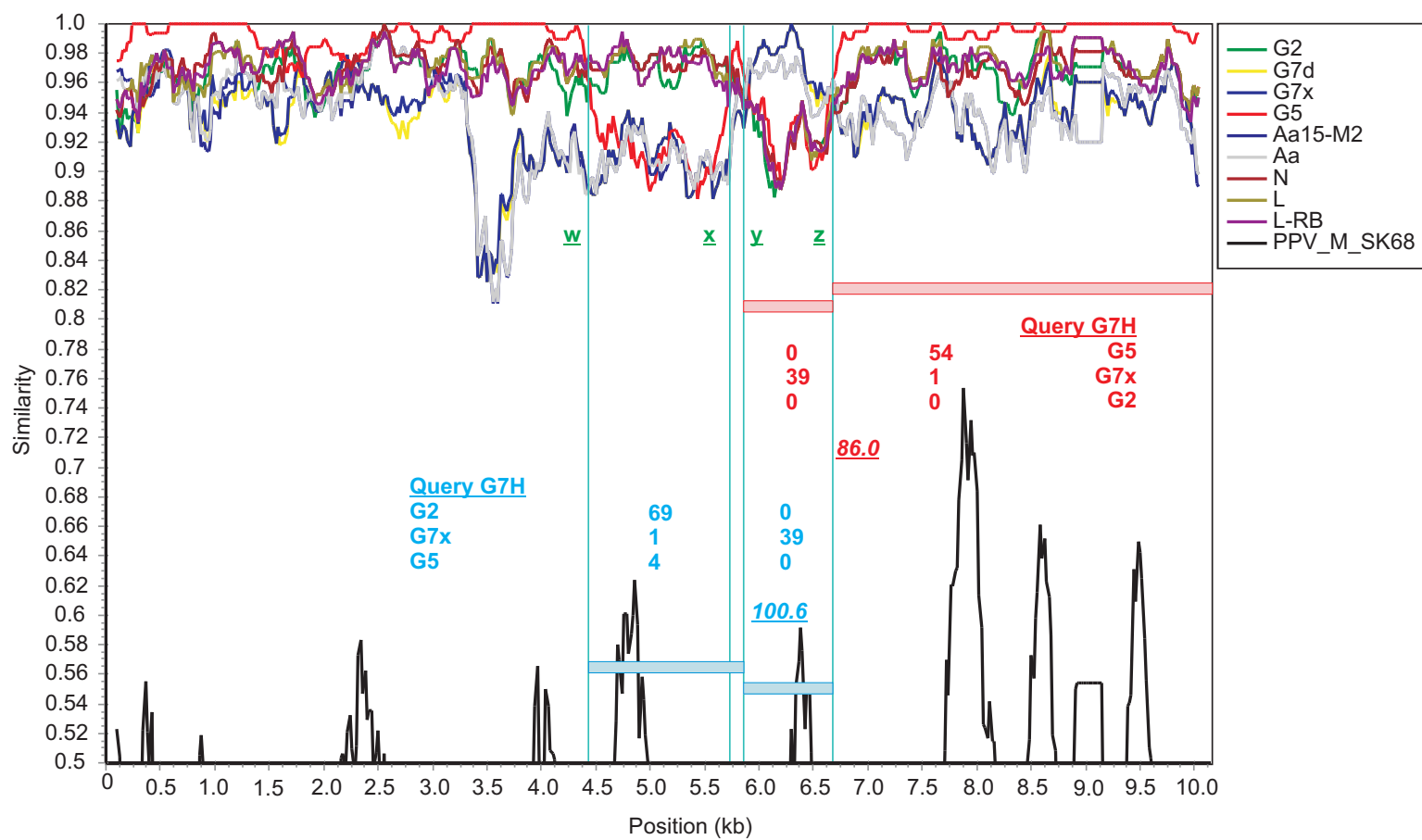

Supplement: Additional File 4 — Supplemental Figure 2. Similarity plots with G7H as the query isolate. Lists of isolates included in the analyses with their corresponding line colors are shown in the legend box. Locations of sites 'w', 'x', 'y', and 'z' are demarcated with vertical lines and the green underlined letters. Regions used for "find sites" analyses are marked with rectangles; names for the query, first and second parental, as well as outgroup isolates, with respective numbers of informative sites, supporting each grouping, and the χ2 values are given for each recombination site in matching colors. [file 1743-422X-5-143-S4.pdf]
