# Supplementary material for: Recombination analysis of Soybean mosaic virus sequences reveals evidence of RNA recombination between distinct pathotypes
Source: Virol J. 2008 Nov 26;5:143. doi: 10.1186/1743-422X-5-143 (PMC2627826; doi:10.1186/1743-422X-5-143)
Supplement: Additional File 6 — Supplemental Table 1. Effect of informative site nucleic acid differences on amino acid composition in analyses of G7f recombination events with remotely related non-SMV potyvirus sequence (PPV) as outgroup. Informative sites that are also found in analyses with Aa as outgroup [see Additional file 7] are given in italics. [file 1743-422X-5-143-S6.pdf]

#	Nucleotide position #		G7f / G2		G7x	
	In G7f	In codon	codon	aa	codon	aa
1	5114	3	GGA	Gly	GGC	Gly
2	5129	3	TTG	Leu	TTA	Leu
3	*5177*	*3*	*GGG*	*Gly*	*GGA*	*Gly*
4	*5180*	*3*	*TTC*	*Phe*	*TTT*	*Phe*
5	*5187*	*1*	*GTC*	*Val*	*ATC*	*Ile*
6	5189	3	GTC	Val	GTT	Val
7	5191	2	AAG	Lys	AGG	Arg
8	*5234*	*3*	*GAC*	*Asp*	*GAT*	*Asp*
9	*6104*	*3*	*GAC*	*Asp*	*GAT*	*Asp*
10	*6113*	*3*	*CCG*	*Pro*	*CCA*	*Pro*
11	*8876*	*3*	*GTA*	*Val*	*GTT*	*Val*
12	*8909*	*3*	*GCT*	*Ala*	*GCC*	*Ala*
13	8945	3	CAG	Gln	CAA	Gln
14	*8963*	*3*	*AAA*	*Lys*	*AAG*	*Lys*
