# Supplementary material for: Recombination analysis of Soybean mosaic virus sequences reveals evidence of RNA recombination between distinct pathotypes
Source: Virol J. 2008 Nov 26;5:143. doi: 10.1186/1743-422X-5-143 (PMC2627826; doi:10.1186/1743-422X-5-143)
Supplement: Additional File 7 — Supplemental Table 2. Effect of informative site nucleic acid differences on amino acid composition in analyses of G7f recombination events with Aa as outgroup. Informative sites that are also found in analyses with non-SMV potyvirus sequence (PPV) as outgroup [see Additional file 6] are given in italics. [file 1743-422X-5-143-S7.pdf]

#	Nucleotide position #		G7f / G2		G7x	
	In G7f	In codon	codon	aa	codon	aa
1	5149	2	ACA	Thr	ATA	Ile
2	5156	3	TGT	Cys	TGC	Cys
3	*5177*	*3*	*GGG*	*Gly*	*GGA*	*Gly*
4	*5180*	*3*	*TTC*	*Phe*	*TTT*	*Phe*
5	*5187*	*1*	*GTC*	*Val*	*ATC*	*Ile*
6	5207	3	ACG	Thr	ACA	Thr
7	*5234*	*3*	*GAC*	*Asp*	*GAT*	*Asp*
8	5246	3	ATT	Ile	ATC	Ile
9	5252	3	CGC	Arg	CGA	Arg
10	6026	3	GAT	Asp	GAC	Asp
11	6059	3	CAA	Gln	CAG	Gln
12	6077	3	AAG	Lys	AAA	Lys
13	*6104*	*3*	*GAC*	*Asp*	*GAT*	*Asp*
14	*6113*	*3*	*CCG*	*Pro*	*CCA*	*Pro*
15	6140	3	AAC	Asn	AAT	Asn
16	8858	3	GTT	Val	GTC	Val
17	8867	3	GGC	Gly	GGT	Gly
18	*8876*	*3*	*GTA*	*Val*	*GTT*	*Val*
19	8882	3	TGC	Cys	TGT	Cys
20	*8909*	*3*	*GCT*	*Ala*	*GCC*	*Ala*
21	*8963*	*3*	*AAA*	*Lys*	*AAG*	*Lys*
22	9008	3	CAC	His	CAT	His
